# Hypoxia and Hormone-Mediated Pathways Converge at the Histone Demethylase KDM4B in Cancer

**DOI:** 10.3390/ijms19010240

**Published:** 2018-01-13

**Authors:** Jun Yang, Adrian L. Harris, Andrew M. Davidoff

**Affiliations:** 1 Department of Surgery, St. Jude Children’s Research Hospital, 262 Danny Thomas Place, Memphis, TN 38105, USA; Andrew.Davidoff@stjude.org; 2 Molecular Oncology Laboratories, Department of Oncology, Weatherall Institute of Molecular Medicine, University of Oxford, Oxford OX3 9DS, UK; aharris.lab@imm.ox.ac.uk

**Keywords:** estrogen receptor alpha, hypoxia-inducible factor 1, KDM4B, endocrine therapy resistance

## Abstract

Hormones play an important role in pathophysiology. The hormone receptors, such as estrogen receptor alpha and androgen receptor in breast cancer and prostate cancer, are critical to cancer cell proliferation and tumor growth. In this review we focused on the cross-talk between hormone and hypoxia pathways, particularly in breast cancer. We delineated a novel signaling pathway from estrogen receptor to hypoxia-inducible factor 1, and discussed the role of this pathway in endocrine therapy resistance. Further, we discussed the estrogen and hypoxia pathways converging at histone demethylase KDM4B, an important epigenetic modifier in cancer.

## 1. Introduction

A solid tumor is a heterogeneous mass that is comprised of not only genetically and epigenetically distinct clones, but also of areas with varying degree of hypoxia that result from rapid cancer cell proliferation that outgrows its blood supply. To survive in hostile hypoxic environments, cancer cells decelerate their proliferation rate, alter metabolism and cellular pH, and induce angiogenesis [1]. These responses of cells to hypoxia are largely coordinated by hypoxia-inducible factors HIF-1 and HIF-2 [2], which drive the expression of a plethora of target genes, such as vascular endothelial growth factor (*VEGF*), carbonic anhydrase IX (*CA9*), and glucose transporter 1 (*Glut-1*) [2]. HIF is a heterodimer composed of an alpha subunit (HIF-1α or HIF-2α) and a β subunit (HIF-1β) [3]. HIF-1β is constitutively expressed, whereas HIF-α is regulated by oxygen availability. Under conditions of normoxia, HIF-α is hydroxylated at conserved prolyl residues by oxygen-dependent prolyl hydroxylases (PHDs), resulting in binding of the von Hippel-Lindau protein (VHL), an E3 ubiquitin ligase, for ubiquitin-mediated degradation [4,5,6]. Under conditions of hypoxia, however, HIFα is stabilized through inhibition of hydroxylation, leading to transactivation of its target genes. Preclinical and clinical studies show that the hypoxia/HIF-1 pathway plays an important role in promoting local tumor invasion and distal metastasis, as well as negatively influencing the responses to radiotherapy and chemotherapy [2,7,8,9,10,11]. The hypoxia/HIF-1 pathway is also involved in immunosuppression and resistance to immunotherapy [12]. In addition to oxygen-mediated degradation of HIFα through VHL, the hypoxia signaling pathway is regulated by a variety of oncogenes (e.g., ERK [13], HER2 [14], mTOR [15], Ras [16,17]) tumor suppressors (e.g., LKB1 [18], PML [19], PTEN [20,21], p53 [22], SDHB [23]), and metabolites (e.g., 2-hydroxyglutarate [24], succinate [25], and fumarate [26]). Here, we focus on the cross-talk between hypoxia and estrogen-mediated pathways, which converge to regulate epigenetic modulators in breast cancer, one of the most common cancers with 450,000 deaths each year worldwide.

## 2. The Cross-Talk between Hypoxia and Estrogen

Fifty years ago, Mirand et al. reported that in rodents, estradiol cyclopentylpropionate (ECP) was able to inhibit erythropoiesis by suppressing the production of erythropoiesis stimulating factor (ESF), which is now known as erythropoietin (EPO) [27], a direct target of hypoxia inducible factor. A subsequent study in 1973 by Gordon et al. further confirmed that estrogen inhibits the production of EPO in female rats exposed to various degrees of hypoxia [28]. Paradoxically, estrogen increases splenic erythropoiesis that is accompanied with elevated plasma EPO levels [29]. Interestingly, the expression of *EPO* mRNA is stimulated by both estradiol and hypoxia in the mouse uterus [30], and the hypoxic induction of *EPO* requires the presence of estradiol [30]. In 1999, Ruohola et al. reported that estradiol caused an increase of the HIF target *VEGF* mRNA in MCF-7 breast cancer cells, which was blocked by antiestrogen ICI 182780, suggesting that the effect was mediated by the estrogen receptor [31]. Subsequent studies further demonstrated the dual regulation of *VEGF* by hypoxia and estrogen [32,33,34]. These data indicate that estrogen and hypoxia pathways are connected. A later study showed that 17-β estradiol attenuates the hypoxic induction of HIF-1α and EPO in Hep3B cells [35]. However, in estrogen receptor-positive breast cancer cells, estrogen induces activation of HIF-1α [34] and co-operates with hypoxia to regulate the expression of a subgroup of genes [36]. Estrogen receptor antagonists (e.g., tamoxifen, raloxifene, or bazedoxifene) all suppress HIF-1α protein accumulation in osteoclast precursor cells [37]. Therefore, estrogen-mediated signaling can either negatively or positively affect the hypoxia pathway in different cellular contexts.

Estrogen receptor alpha (ERα) is an estrogen-dependent nuclear transcription factor that is not only critical for mammary epithelial cell division, but also breast cancer progression [38,39]. Despite the multiple molecular subtypes that have been classified based on transcriptomic and genetic features [40], ERα is one of the most important biomarkers directing breast cancer treatment. It is recommended that all patients with ERα positivity should have adjuvant endocrine therapy. ERα is expressed in approximately 70% of breast tumors [41], the majority of which depend on estrogen signaling, thereby providing the rationale for using anti-estrogens as adjuvant therapy to treat breast cancer [42]. Endocrine therapy drugs for breast cancer include selective ER modulators, such as tamoxifen, antagonists such as fulvestrant, and aromatase inhibitors such as anastrozole. Tamoxifen is a first-generation selective ER modulator (SERM) and has been widely used in breast cancer prevention and treatment [42]. It antagonizes ERα function in breast cancer cells by competing with estrogen for ERα binding while preserving its activating and estrogen-like functions in the bone [43]. Although now replaced by aromatase inhibitors (AI) as first-line treatment in post-menopausal women, tamoxifen still remains important in premenopausal breast cancer and after failure of AIs. The antagonist fulvestrant leads to ERα protein degradation [44], while aromatase inhibitors block the conversion of androgens to estrogens thereby reducing overall estrogen levels [45]. The application of endocrine therapies has led to a significant reduction in breast cancer mortality [46]. However, not all ER-positive patients respond to endocrine therapies and nearly all women with advanced cancer will eventually die from metastatic disease [47,48], as resistance often develops [49]. Many mechanisms have been proposed to account for endocrine therapy resistance [50,51], including loss of ERα expression or expression of truncated ER isoforms, posttranslational modification of ERα, deregulation of ERα co-activators, and increased receptor tyrosine kinase signaling. Recent studies further indicate that somatic ERα mutation [52,53], as well as genomic amplification of distant ER response elements [54] could contribute to hormone therapy resistance. Hypoxia is also involved in endocrine therapy resistance. Clinical studies have shown that HIF-1α expression is associated with an aggressive phenotype of breast cancer, i.e., large tumor size, high grade, high proliferation rate, and lymph node metastasis [55]. Increased HIF-1α is also associated with ERα positivity [55], whilst HIF-1β, the partner of HIF-1α, has been shown to function as a potent co-activator of ER-dependent transcription [56]. Importantly, HIF-1α protein expression was associated with tamoxifen resistance in neoadjuvant, primary therapy of ERα-positive breast cancers [57], as well as resistance to chemoendocrine therapy [58].

**Figure 1:**
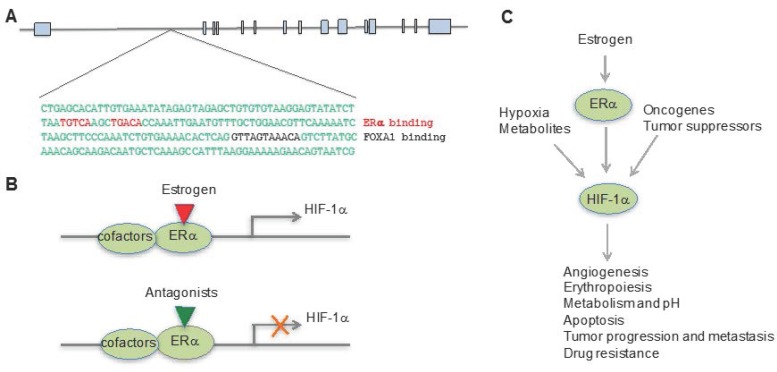
Estrogen pathway directly drives HIF-1α expression. (**A**) *HIF-1α* gene bears a canonical estrogen receptor binding element (ERE), with a FOXA1 binding site downstream of ERE; (**B**) When ERα is bound by its ligand it drives the expression of HIF-1α. However, ERα antagonists block the expression of HIF-1α; (**C**) The pathways mediated by hypoxia, estrogen, metabolites, and cancer genes converge on HIF-1α, which drives a plethora of genes that are involved in multiple biological processes, cancer progression, and therapeutic resistance.

The exact nature of the relationship between hypoxia and estrogen pathways was a puzzle until our recent findings showing that the *HIF-1α* gene is a direct target of ERα [59]. In this study, we analyzed the global gene expression profile in response to hypoxia and the ERα antagonist fulvestrant and found a subgroup of genes that were dually responsive to the hormone and to oxygen. These genes were upregulated by hypoxia but the ERα antagonist fulvestrant significantly reduced their expression. These data were consistent with previous studies that showed some genes, such as *KDM4B*, *STC2*, and *VEGF*, bear both a hypoxia response element and estrogen response element [60,61,62,63,64,65,66]. Most interestingly, we found that ERα signaling directly regulates HIF-1α expression. When MCF7 cells were grown without estrogen and then placed in hypoxia or treated with the hypoxia mimetic deferoximine, estradiol greatly enhanced HIF-1α expression and this was reversed by fulvestrant and ERα depletion. By analyzing the *HIF-1α* genomic sequence that bears 15 exons and 14 introns, we identified a canonical estrogen response element (ERE) located in the first intron (Figure 1A). Interestingly, there is also a FOXA1 binding site that is 64 nucleotides downstream of ERE, further supporting it as a bona fide ERα binding element, because FOXA1 is a pioneer factor that facilitates ERα recruitment [67]. Actually, one study has shown that overexpression of FOXA1 in ER-positive breast cancer cell lines promotes resistance to tamoxifen and to estrogen deprivation [68]. We further validated our findings by chromatin immunoprecipitation-PCR and a luciferase reporter assay, showing that ERα directly binds to this locus, driving *HIF-1α* gene expression. This finding not only explains the early findings that estrogen and hypoxia pathways crosstalk, but also indicates that overactive HIF-1α function may partially compensate for estrogen signaling when ERα function is compromised, such as in the circumstance of hormone therapy, leading to hormone therapy resistance (Figure 1B,C).

## 3. The Hypoxia and Estrogen Signaling Pathways Converge on Histone Demethylases

Genetic abnormalities that drive tumorigenesis are usually coupled with epigenetic alterations that engage multiple important biological processes such as DNA replication, DNA repair, and gene expression [69,70,71,72,73]. One such aberrant chromatin modification is histone lysine methylation [69,70], which was believed to be irreversible until the discovery of lysine-specific demethylase 1 (LSD1) [74]. Subsequent studies identified another family of histone demethylases, the Jumonji C (JmjC) domain–containing demethylases [75], which require iron and 2-oxoglutarate (2-OG) for their activities. The JmjC histone lysine demethylase family (KDMs) is composed of 17 members and is responsible for reversing most of the histone methyl marks in the human genome. Dysregulated histone lysine methylation is commonly seen in various cancers [76], which is consistent with observed genetic alterations and/or dysregulation of histone methyltransferases and KDMs [75,77,78,79,80,81]. Interestingly, we and others have shown that many JmjC histone demethylases are hypoxia-inducible [61,82], some of which, including KDM3A [60,61,83], KDM4B [60,61,63], and KDM4C [84], are direct targets of HIFs.

**Figure 2:**
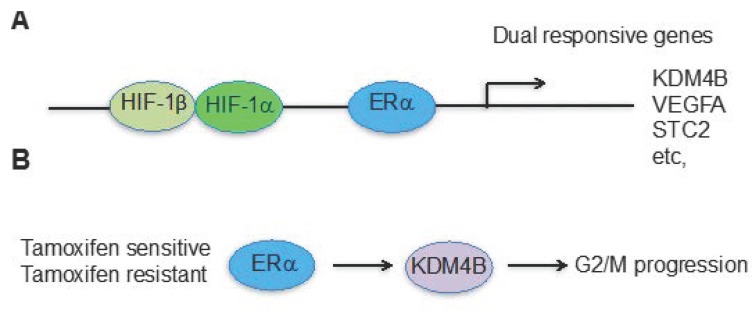
Hypoxia and estrogen pathways converge at KDM4B for cancer cell proliferation in ERα positive breast cancer. (**A**) KDM4B is one of the genes responsive to both estrogen and hypoxia-mediated pathways; (**B**) Regardless of endocrine therapy resistance, ERα drives KDM4B expression, which is required for G2/M phase progression.

The KDM4 subfamily of histone demethylases consists of four members. KDM4A, KDM4B, and KDM4C share high sequence homology in their catalytic domains, and they remove methyl groups from H3K9me2/me3 and H3K36me2/me3 [75]. KDM4A-4C members also bear other similar functional domains that include two PHD domains and two Tudor domains. However, KDM4D is less conserved and removes methyl groups only from H3K9me2/me3. KDM4B plays important roles in the self-renewal of embryonic stem cells and the conversion of induced pluripotent stem cells [85,86], and is linked to many forms of cancer [87]. KDM4B is amplified in medulloblastoma [88] and malignant peripheral nerve sheath tumors [89], and is overexpressed in many other cancers [90,91,92]. KDM4B regulates the expression of key oncogenes, such as *C-MYC* [93,94,95,96] and *CDK6* [97], and is involved in cancer invasiveness, metastasis, and therapeutic resistance [98,99,100]. Interestingly, KDM4B is a direct target of p53, exerting its DNA repair function in response DNA damage [101,102,103]. Recently, we showed that KDM4B is involved in neuroblastoma growth and tumor maintenance [104]. The expression of KDM4B was highly correlated with that of the *MYCN* oncogene in neuroblastoma, and it formed a complex with N-Myc protein, thereby facilitating its function by maintaining low levels of repressive H3K9me2/me3 marks at Myc-binding sites. In breast cancer we have shown that HIF-1α and ERα can coordinate expression of genes, such as KDM4B, whose expression is driven by both ERα and HIF-1α and epigenetically regulates the G2/M phase of cell cycle progression in breast cancer cells [63] and other cancer cell lines (unpublished data), as the expression of several key cell cycle genes is correlated with changes in the KDM4B substrate, H3K9me3 [63,104]. Similar to other dual responsive genes such as *VEGF* and *STC2* that are regulated by both estrogens and hypoxia, the genomic locus of KDM4B bears both HIF-1α and ERα binding elements [60,63] (Figure 2A). The cross-talk between HIF-1α and ERα converges at KDM4B, which is important for cell cycle progression and tumor growth in ER positive breast cancer [63,93]. Importantly, in endocrine therapy-resistant breast cancer cells, the regulation of KDM4B by HIF-1α and ERα is intact and KDM4B is still required for G2/M phase progression [59] (Figure 2B). In addition, KDM4B is not only required for enhancing androgen receptor (AR) transcriptional activity through histone modification, but it also enhances AR protein stability via inhibition of AR ubiquitination [105], demonstrating the functional connection between AR and KDM4B in prostate cancer. Therefore, HIF-1α plays an important role in modulating anti-androgen responses via KDM4B in prostate cancer.

## 4. Future Prospects

Many transcriptional factors, such as Myc, ERα, and AR, exert oncogenic functions to drive cancer cell proliferation. Directly targeting these oncogenic transcription factors is either technically challenging or leads to therapeutic resistance. Therefore, new approaches need to be developed to overcome these obstacles. Transcription factors need to complex with other cofactors to drive gene expression and many of these cofactors are histone modifiers. Thus, development of small molecules to target the histone modifiers, such as KDM4B, may provide an opportunity to enhance the efficacy of standard chemotherapeutics or to overcome drug resistance. Recently, efforts have been made by us and other groups to identify and develop KDM4B inhibitors for cancer treatment [106,107,108,109]. By using a chemoinformatics in combination with high-content imaging approach we identified ciclopirox as a novel histone demethylase inhibitor. Ciclopirox targeted KDM4B, inhibited Myc signaling, resulting in suppression of neuroblastoma cell viability and tumor growth associated with an induction of differentiation [107]. We also found that MCF7 cells (ERα-positive) were much more sensitive to MDA-MB-231 cells (ERα-negative) (Jun Yang, St Jude Children’s Research Hospital, Memphis, TN, USA. unpublished data), suggesting ERα-positive breast cancer cells are more addicted to KDM4B. Chu et al. identified a KDM4B inhibitor that significantly blocked the viability of cultured prostate cancer cells, which was accompanied by transcriptional silencing of growth-related genes, a substantial portion of which were AR-responsive [106]. Recently, a more potent and selective KDM4 inhibitor was developed by Cellgene [108,109], which was efficacious in breast and colon cancer models. Although whether these KDM4B inhibitors are able to reverse endocrine therapy resistance needs to be tested, we believe specific and potent KDM4B inhibitors hold a promise for overcoming endocrine therapy resistance to breast cancer and prostate cancer.
